# p53-Mediated Indirect Regulation on Cellular Metabolism: From the Mechanism of Pathogenesis to the Development of Cancer Therapeutics

**DOI:** 10.3389/fonc.2022.895112

**Published:** 2022-05-30

**Authors:** Chen-Yun Wang, Chi-Hong Chao

**Affiliations:** ^1^ Institute of Molecular Medicine and Bioengineering, National Yang Ming Chiao Tung University, Hsinchu, Taiwan; ^2^ Center For Intelligent Drug Systems and Smart Bio-devices (IDS2B), National Yang Ming Chiao Tung University, Hsinchu, Taiwan; ^3^ Department of Biological Science and Technology, National Yang Ming Chiao Tung University, Hsinchu, Taiwan

**Keywords:** wild-type p53, mutant p53, indirect regulation, metabolism, cancer treatment, synthetic lethality

## Abstract

The transcription factor p53 is the most well-characterized tumor suppressor involved in multiple cellular processes, which has expanded to the regulation of metabolism in recent decades. Accumulating evidence reinforces the link between the disturbance of p53-relevant metabolic activities and tumor development. However, a full-fledged understanding of the metabolic roles of p53 and the underlying detailed molecular mechanisms in human normal and cancer cells remain elusive, and persistent endeavor is required to foster the entry of drugs targeting p53 into clinical use. This mini-review summarizes the indirect regulation of cellular metabolism by wild-type p53 as well as mutant p53, in which mechanisms are categorized into three major groups: through modulating downstream transcriptional targets, protein-protein interaction with other transcription factors, and affecting signaling pathways. Indirect mechanisms expand the p53 regulatory networks of cellular metabolism, making p53 a master regulator of metabolism and a key metabolic sensor. Moreover, we provide a brief overview of recent achievements and potential developments in the therapeutic strategies targeting mutant p53, emphasizing synthetic lethal methods targeting mutant p53 with metabolism. Then, we delineate synthetic lethality targeting mutant p53 with its indirect regulation on metabolism, which expands the synthetic lethal networks of mutant p53 and broadens the horizon of developing novel therapeutic strategies for p53 mutated cancers, providing more opportunities for cancer patients with mutant p53. Finally, the limitations and current research gaps in studies of metabolic networks controlled by p53 and challenges of research on p53-mediated indirect regulation on metabolism are further discussed.

## Introduction

During the 40 years of discovery, *TP53*, encoding a transcription factor known as the guardian of the genome, has been well characterized as a pivotal tumor suppressor. Upon various stress signals, including DNA damage, metabolic stress, and induction of oncogenes, p53 is released from its core negative regulator MDM2 and activated through multiple post-translational modifications (PTMs), which subsequently results in the upregulation or downregulation of genes involved in DNA repair, cell-cycle arrest, senescence, and apoptosis. Furthermore, p53 has been identified as an important regulator of stemness, autophagy, redox homeostasis, cellular metabolism, as well as tumor microenvironments (TMEs) (1–3).

Being the most frequently mutated tumor suppressor gene in human cancers, *TP53* mutation exists in more than 50% of human cancers. *TP53* mutates variably in different types or subtypes of cancer (4), and the majority of *TP53* mutations are missense mutations, a single-base substitution located in the DNA-binding domain (DBD), giving rise to a full-length p53 protein (5). The existence of hotspot mutations, which account for almost 30% of all the missense mutations in *TP53*, may confer maximal benefits on tumor cells (6). These mutations are classified into two main categories: DNA-contact mutations abrogate residues directly involved in DNA binding, such as R273H, while structural or conformational mutations disrupt the structure of the DBD, such as R175H (3). Typically, oncogenic effects of *TP53* mutations might be exerted through loss of function (LOF) of wild-type p53 (WTp53), dominant-negative effect (DNE) over WTp53, and gain-of-function (GOF) independent of WTp53, which are not mutually exclusive (7, 8). Despite losing sequence-specific DNA binding (SSDB) activity, mutant p53 (MTp53) can regulate gene expression through both direct and indirect mechanisms (7, 9, 10). Direct binding of MTp53 to DNA can be achieved by DNA structure-selective binding (DSSB), in which MTp53 recognizes its target genes by selective binding to DNA secondary structures. On the other hand, MTp53 associates with various transcription factors to act as a transcriptional repressor or transcription cofactor. Moreover, cooperation between MTp53 and the SWI/SNF chromatin remodeling complex contributes to over 40% of MTp53-regulated gene expression (11). MTp53 also exerts its oncogenic functions *via* modulating non-coding RNAs (ncRNAs) (12). Taken together, all these facts reveal a significant contribution of the indirect mechanisms to MTp53-mediated biological effects.

As previously mentioned, p53 has emerged as a critical modulator of cellular metabolism. Actually, p53-mediated metabolic activities have been reported to involve in the development of several human diseases, including diabetes, ischemia, neurodegeneration, as well as cancer (13), which we will focus on in this review. In addition to inducing or repressing the expression of transcriptional target genes associated with p53-mediated metabolic pathways and directly interacting with metabolic enzymes to activate or inhibit their activities, p53 also regulates metabolism *via* indirect mechanisms. The indirect mechanisms which we define here are categorized into three groups. First, p53 regulates metabolic genes through modulating its direct targets, such as microRNAs (miRNAs) and long non-coding RNAs (lncRNAs). Second, p53 activates or suppresses the expression of metabolic genes through interacting with other transcription factors. Third, p53 controls metabolism by affecting signaling pathways. These indirect mechanisms scale up the number of metabolism-associated genes regulated by p53, extend the influence of p53 to a wide variety of metabolic pathways, and expand the p53 regulatory networks of metabolism, further highlighting p53 as a master regulator of metabolism. The critical role of p53-mediated indirect regulation on metabolism in tumorigenesis and tumor progression is evident, for example, in our previous study that in spite of several direct transcriptional targets of p53 involved in mitochondrial respiration, restoration of the decreased oxidative phosphorylation (OXPHOS) caused by MTp53 could be achieved through ectopic expression of miR-200c, a downstream direct transcriptional target of p53, which enhances phosphoenolpyruvate carboxykinase 2 (PCK2) expression *via* downregulating ZEB1/BMI1 (14). Moreover, restoring miR-200c expression leads to suppression of tumor growth, whereas interference of PCK2, the key enzyme linking the TCA cycle and glycolysis (15), counteracts the miR-200c-mediated tumor-suppressing effect, which accentuates the vital role of the indirect mechanisms accounting for p53-regulated cellular metabolism, and provides a functional link between p53-regulated metabolism and p53-mediated biological functions. Furthermore, p53 is generally considered an important metabolic sensor by positioning at the center of several signaling pathways coordinating cellular metabolism (16). Notably, direct and indirect regulation of metabolism by p53 are not mutually exclusive but overlapped to some extent. This is exemplified by hexokinase 2 (HK2), the first rate-limiting enzyme of glycolysis, which is both a direct transcriptional target of p53 (17) and an indirect metabolic target of p53 through miR-34a (18) and miR-143 (19). Interestingly, glucose transporter 1 (GLUT1), transcriptionally repressed by p53 (20) is an indirect target of MTp53 through modulation of the RhoA/ROCK signaling pathway (21), indicating the flexibility of indirect mechanisms underlying p53-mediated metabolic activities.

**Figure 1:**
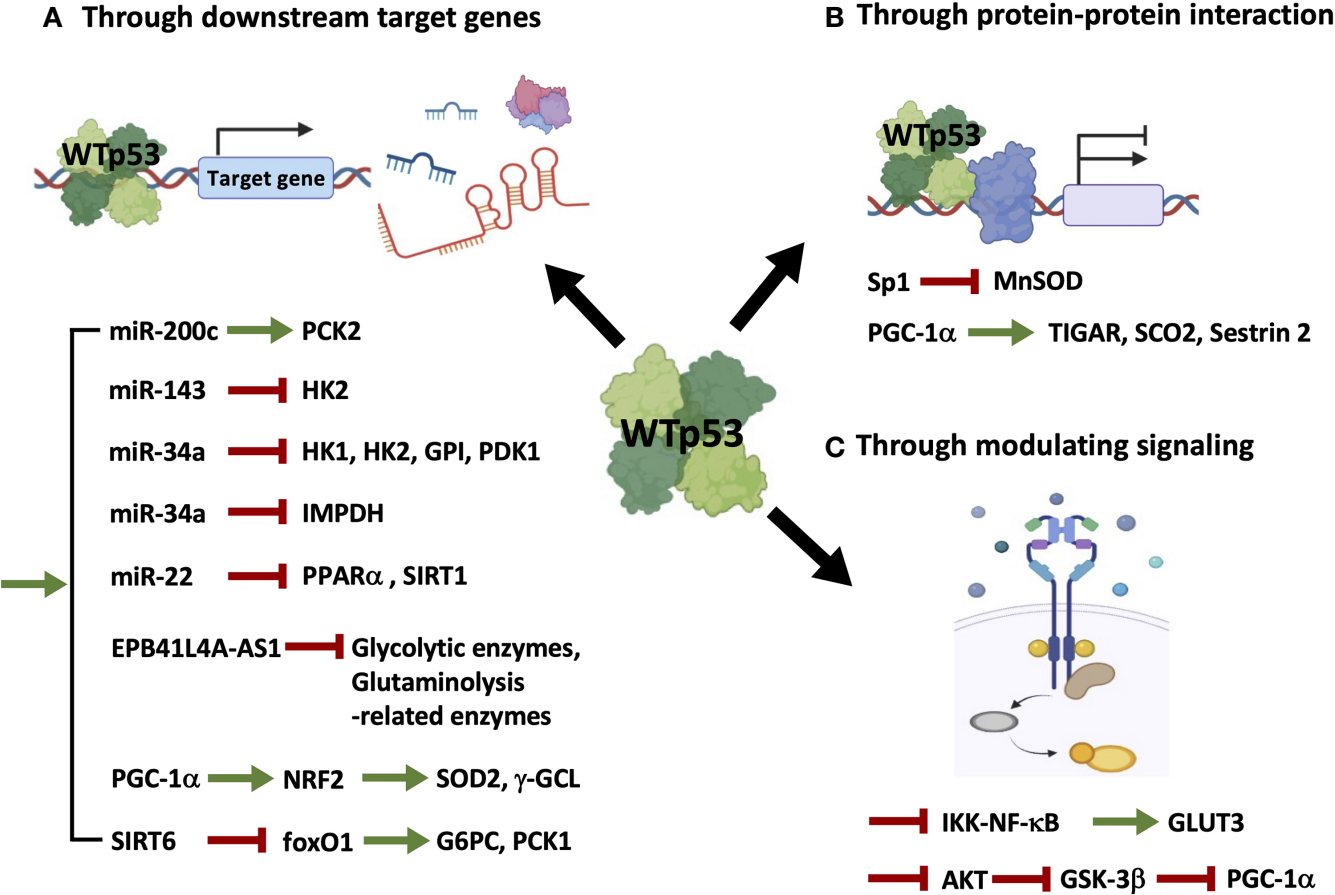
Wild-type p53 regulates cellular metabolism through indirect mechanisms. WTp53 indirectly regulates metabolism through inducing its direct targets, including miRNAs, lncRNAs, and proteins **(A)**, associating with other transcription factors **(B)**, or modulating signaling pathways **(C)**.

**Figure 2:**
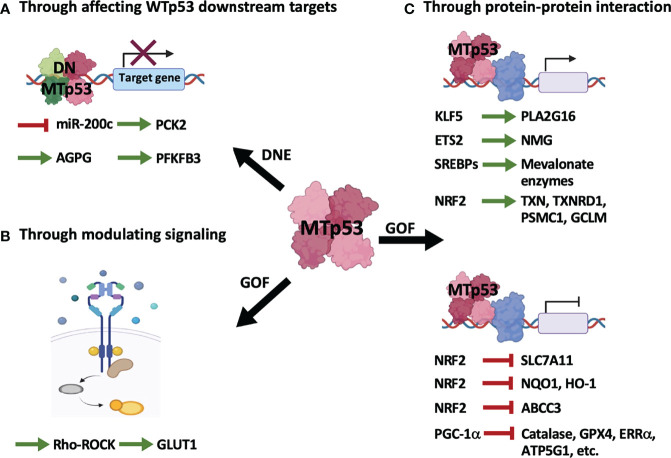
Mutant p53 regulates cellular metabolism through indirect mechanisms. MTp53 indirectly regulates metabolism by its dominant-negative effect (DNE) over WTp53 **(A)**, and the gain-of-function (GOF) properties encompassing modulation of signaling pathways **(B)** or interaction with other transcription factors **(C)**.

Transcriptional targets of p53 in regulating metabolism have been delineated in widespread literature, especially in a review article that gives a clear summary of direct target genes activated or repressed by p53 in every aspect of metabolic pathways (16). Considering that the indirect mechanisms seem to play a critical role in contributing to p53-mediated control of cellular metabolisms and other biological functions, and a growing number of metabolic-associated genes and proteins have been reported to be indirectly modulated by p53, in the present work, we will briefly summarize the indirect regulation of cellular metabolism by WTp53 (**Figure 1** and **Table 1**) and MTp53 (**Figure 2** and **Table 2**), respectively.

**Table 1:** Metabolic targets indirectly regulated by wild-type p53.

p53 Status	Targets	Mechanism of Regulation	Metabolic Effect	Biological Consequence	Ref.
(A) Through transcriptionally regulating downstream target genes					
WTp53	Phosphoenolpyruvate carboxykinase 2 (PCK2)	Induce miR-200c to upregulate expression.	Increased OXPHOS.	p53 mutation facilitates cancer stemness.	(14)
WTp53	Hexokinase 2 (HK2)	Induce miR-143, which facilitates degradation of HK2 mRNA.	Decreased aerobic glycolysis.	Loss of p53 leads to *Pten/p53*-deficiency-driven proliferation, transformation, and *in vivo* tumor growth.	(19)
WTp53	Hexokinase 1 (HK1), HK2, Glucose-6-phosphate isomerase (GPI), Pyruvate dehydrogenase kinase 1 (PDK1)	Induce miR-34a to downregulate expression.	Decreased glycolysis and increased mitochondrial respiration.	Not applicable.	(18)
WTp53	Inosine 5’-monophosphate dehydrogenase (IMPDH)	Induce miR-34a to downregulate expression.	Decreased GTP biosynthesis (purine synthesis).	p53 represses GTP-dependent Ras signaling pathway.	(22)
WTp53	Peroxisome proliferator-activated receptor-α (PPARα), NAD^+^-dependent histone deacetylase sirtuin 1 (SIRT1)	Induce miR-22 to downregulate expression.	Decreased FAO.	Blockade of this signaling pathway ameliorates high-fat diet (HFD)-induced hepatic steatosis.	(23)
WTp53	Glycolytic enzymes such as HK1 and pyruvate kinase M2 (PKM2); Glutaminolysis-related enzymes such as alanine-serine-cysteine transporter type 2 (ASCT2) and Glutaminase 2 (GLS2)	Induce the lncRNA EPB41L4A-AS1 to downregulate expression through modulating the VHL/HIF-1α pathway and the VDAC1/ATF4 pathway, respectively	Decreased glucose uptake, glycolysis, and lactate production. Decreased glutaminolysis.	Depletion of EPB41L4A-AS1 largely increases the anti-tumor effect of glutaminase inhibitors.	(24)
WTp53	Manganese superoxide dismutase (MnSOD or SOD2), γ-glutamylcysteine ligase (γ-GCL)	Transactivate PGC-1α to upregulate expression through NRF2.	Decreased ROS.	Blockade of the p53-PGC-1α-NRF2 pathway increases ROS and cell death.	(25)
WTp53	Glucose-6-phosphatase (G6PC), Phosphoenolpyruvate carboxykinase 1 (PCK1)	Transactivate SIRT6 to cause deacetylation and nuclear exclusion of foxO1, which is the inducer of G6PC and PCK1.	Decreased gluconeogenesis.	p53 decreases the recovery of murine blood glucose levels induced by pyruvate.	(26)
(B) Through protein-protein interaction					
WTp53	Manganese superoxide dismutase (MnSOD or SOD2)	Associate with Sp1 to inhibit transcription.	Not applicable.	Not applicable.	(27)
WTp53	TP53-induced glycolysis regulatory phosphatase (TIGAR), Synthesis of cytochrome C oxidase 2 (SCO2), Sestrin 2	Recruit PGC-1α to upregulate expression.	Decreased ROS.	p53 binds to PGC-1α to promote cell-cycle arrest and ROS clearance at early periods of glucose starvation.	(28)
(C) Through modulating signaling pathways					
WTp53	Glucose transporter 3 (GLUT3)	Inhibit the IKK-NF-κB pathway, which activates GLUT3.	Decreased glycolysis and lactate production.	p53 deficiency leads to oncogene-induced cell transformation.	(29)
WTp53	Peroxisome proliferator-activated receptor γ coactivator-1α (PGC-1α)	Inhibit AKT and activate GSK-3β to promote degradation through the ubiquitin-proteasome system.	Decreased mitochondrial function.	Knockdown of PGC-1α synergizes with cisplatin to promote apoptosis and inhibit tumor growth.	(30)

**Table 2:** Metabolic targets indirectly regulated by mutant p53.

p53 Status	Targets	Mechanism of Regulation	Metabolic Effect	Biological Consequence	Ref.
(A) Dominant-negative effect: Through affecting WTp53 downstream targets					
MTp53 (R175H, R249S, R273H, R280K)	PCK2	Downregulate WTp53-induced miRNA miR-200c, which increases PCK2 expression through inhibiting ZEB1 and BMI1.	Decreased OXPHOS.	Downregulation of PCK2 by MTp53 through the miR-200c-ZEB1/BMI1 axis facilitates cancer stemness.	(14)
MTp53 (V272M, R110L + E326K)	Fructose-2,6-biphosphatase 3 (PFKFB3)	Rescue WTp53-repressed lncRNA AGPG to prevent ubiquitination and stabilize PFKFB3.	Enhanced glycolysis.	Upregulation of AGPG by MTp53 promotes cell proliferation and *in vivo* tumor growth.	(31)
(B) Gain-of-function: Through modulating signaling pathways					
MTp53 (R175H, R248Q, R273H)	Glucose transporter 1 (GLUT1)	Activate the RhoA/ROCK signaling, which induces GLUT1 translocation to the plasma membrane.	Enhanced glycolysis.	Knocking down GLUT1 abolishes MTp53-promoted anchorage-independent growth and xenograft tumor growth.	(21)
(C) Gain-of-function: Through protein-protein interaction					
MTp53 (R273H, R248W)	Phospholipase A2, group XVI (PLA2G16)	Associate with KLF5 to activate transcription.	Increased glycolysis.	High PLA2G16 predicts a poor prognosis. Knockdown of PLA2G16 impairs proliferation, anchorage-independent growth, and tumor growth.	(32)
MTp53 (R249S, R273L, R280K, R248W)	Nucleotide metabolism genes (NMG)	Associate with ETS2 to activate transcription.	Increased dNTP and rNTP pools.	NMG expression contributes to invasion and metastatic potential.	(33)
MTp53 (R175H, R248Q, R273H, R248W, G245S, R280K)	Mevalonate pathway enzymes such as HMG-CoA reductase (HMGCR)	Associate with SREBPs to activate transcription.	Elevated activity of the mevalonate pathway.	Supplementing metabolites produced by the mevalonate pathway reverses the phenotypic reversion of disrupted acinar formation caused by the KD of MTp53.	(34)
MTp53 (R175H, R280K)	NRF2 target genes Activated: Thioredoxin (TXN), Thioredoxin reductase 1 (TXNRD1), Proteasome 26S subunit ATPase 1 (PSMC1), Glutamate-cysteine ligase modifier subunit (GCLM) Inhibited: Heme oxygenase 1 (HO-1), Solute carrier family 7 member 11 (SLC7A11), ATP binding cassette subfamily C member 3 (ABCC3)	Associate with NRF2 to activate or inhibit transcription.	Not applicable.	Differential regulation of NRF2 targets by MTp53 contributes to cell survival and migration under oxidative stress. MTp53-activated NRF2 targets are correlated with poor prognosis.	(35)
MTp53 (C277F, R248Q, R248W, G266E, R175H, R273H)	SLC7A11	Bind to and interfere with NRF2 to inhibit transcription.	Depleted glutathione and increased ROS.	A low level of SLC7A11 sensitizes cancer cells with MTp53 to APR-246, which induces oxidative stress.	(36)
MTp53 (R273H)	NAD(P)H quinone dehydrogenase (NQO1), HO-1	Interfere with NRF2 to inhibit transcription.	Increased ROS.	MTp53-mediated reduction of phase 2 detoxifying enzymes promotes cell survival following oxidative damage.	(37)
MTp53 (R175H, R273H)	PGC-1α target genes such as Catalase, Glutathione peroxidase 4 (GPX4), Estrogen-related receptor α (ERRα), ATP synthase lipid-binding protein (ATP5G1)	Associate with PGC-1α to inhibit its function.	R72-MTp53 shows increased OXPHOS.	Tumor cells with R72-MTp53 have greater migration, invasion, and metastatic ability than tumor cells with P72-MTp53.	(38)

## Wild-Type p53-Mediated Indirect Regulation on Metabolism

As illustrated in **Figure 1** and summarized in **Table 1**, mechanisms accounting for WTp53-mediated indirect regulation of metabolism can be categorized into three major types: through transcriptionally regulating downstream target genes (**Figure 1A** and **Table 1A**), through protein-protein interactions (**Figure 1B** and **Table 1B**), and through modulating signaling pathways (**Figure 1C** and **Table 1C**).

(A). WTp53 indirectly regulates the expression of metabolism-related genes by modulating its direct targets (**Figure 1A** and **Table 1A**). In our previous study (14), we demonstrate that miR-200c, a WTp53 target negatively regulating epithelial-mesenchymal transition (EMT) and stemness (39), promotes OXPHOS in basal-like breast cancer (BLBC) cells by inhibiting the downstream targets, ZEB1 and BMI1, which subsequently activates PCK2 expression. In prostate cancers, loss of p53 compromises p53-induced miR-143, facilitating degradation of HK2 mRNA, which is required for *Pten/p53*-deficiency-driven aerobic glycolysis, proliferation, transformation, and *in vivo* tumor growth (19). In another study, induction of miR-34a by p53 downregulates hexokinase 1 (HK1), HK2, glucose-6-phosphate isomerase (GPI), and pyruvate dehydrogenase kinase 1 (PDK1), potentiating mitochondrial respiration and decreasing glycolysis (18). In addition to regulating glucose metabolism, p53-induced miR-34a also represses the expression of inosine 5’-monophosphate dehydrogenase (IMPDH), the rate-limiting enzyme of GTP biosynthesis, leading to decreased Ras signaling (22). In the mouse model of diet-induced obesity (DIO)-related hepatic steatosis, activation of the hepatic cannabinoid-1 receptor (CB_1_R) induces expression of miR-22 through modulating the transcriptional activity of p53, which disturbs peroxisome proliferator-activated receptor-α (PPARα) and NAD^+^-dependent histone deacetylase sirtuin 1 (SIRT1), leading to decreased fatty acid oxidation (FAO) and increased fat accumulation in the liver (23). The lncRNA EPB41L4A-AS1, a transcriptional target of WTp53, decreases glycolysis and glutaminolysis *via* interacting with histone deacetylase 2 (HDAC2), in which interference of EPB41L4A-AS1 sensitizes tumor cells to glutaminase inhibitor (24). Aside from modulating non-coding RNAs, p53 transactivates peroxisome proliferator-activated receptor γ coactivator-1α (PGC-1α), the master regulator of mitochondrial biogenesis and function (40), upon glutathione (GSH) depletion, which induces antioxidant response through nuclear factor E2-related factor 2 (NRF2)-mediated expression of manganese superoxide dismutase (MnSOD or SOD2) and γ-glutamylcysteine ligase (γ-GCL) (25). Furthermore, p53 inhibits the expression of the rate-limiting enzymes of gluconeogenesis, glucose-6-phosphatase (G6PC) and phosphoenolpyruvate carboxykinase 1 (PCK1), through transactivating NAD^+^-dependent histone deacetylase sirtuin 6 (SIRT6), which subsequently causes deacetylation and nuclear exclusion of forkhead box protein O1 (foxO1), the inducer of G6PC and PCK1 (26).
(B). WTp53 indirectly regulates metabolism through a protein-protein interaction with other transcription factors (**Figure 1B** and **Table 1B**). For example, p53 associates with the transcription factor, specificity protein 1 (Sp1), to repress transcription of MnSOD (27), an antioxidant enzyme differentially expressed in cancers (41). Interestingly, in addition to transcriptional control, p53 recruits PGC-1α to modulate its transactivation activity at early periods of metabolic stress, as indicated by upregulation of the proarrest and metabolic genes, including TP53-induced glycolysis regulatory phosphatase (TIGAR), synthesis of cytochrome C oxidase 2 (SCO2), and sestrin 2, which induces cell-cycle arrest and reactive oxygen species (ROS) clearance (28).
(C). WTp53 modulates signaling pathways to affect cellular metabolism (**Figure 1C** and **Table 1C**). An example of this is the study from Kawauchi et al. in which p53 deficiency in mouse embryonic fibroblasts (MEFs) leads to activation of the IKK-NF-κB pathway, which transactivates the *Glut3* gene, and increases aerobic glycolysis and lactate production, contributing to oncogene-induced cell transformation (29). In contrast to transactivating or recruiting PGC-1α to serve as a coactivator, p53 destabilizes PGC-1α through activating the ubiquitin-proteasome system, mediated by AKT/GSK-3β-dependent phosphorylation of PGC-1α, which impairs mitochondrial function and increases chemosensitivity of non-small cell lung cancer (NSCLC) (30).

## Mutant p53-Mediated Indirect Regulation on Metabolism

As illustrated in **Figure 2** and summarized in **Table 2**, MTp53 indirectly regulates metabolism either by disturbing WTp53 function (**Figure 2A** and **Table 2A**) or by exerting its gain-of-function properties (**Figures 2B, C** and **Tables 2B, C**).

(A). MTp53-mediated metabolic reprogramming from perturbation of WTp53 downstream targets (**Figure 2A** and **Table 2A**) can be exemplified by our previous research that MTp53 attenuates OXPHOS through downregulating miR-200c, the positive regulator of PCK2, which facilitates cancer stemness in BLBC (14). In our study, the MTp53-exerted dominant-negative effect impedes the expression of miR-200c, which upregulates ZEB1 and BMI1 and subsequent downregulates PCK2. PCK2 deficiency not only leads to decreased OXPHOS in normal mammary epithelial cells but also compromises the increased OXPHOS by restoration of miR-200c in BLBC cells (14). Another example is a recent study that upregulation of the lncRNA Actin Gamma 1 Pseudogene (AGPG) by p53 deficiency leads to stabilization of fructose-2,6-biphosphatase 3 (PFKFB3), contributing to enhanced glycolysis, proliferation, and tumor growth (31).
(B). Independent of WTp53, the gain-of-function of MTp53 has a profound effect on various metabolic pathways. Similar to WTp53, MTp53 also regulates metabolism by affecting signaling pathways (**Figure 2B** and **Table 2B**). This is evidenced in a study that MTp53-boosted glycolysis promotes tumorigenesis through activation of the RhoA/ROCK signaling, subsequently inducing actin polymerization and translocation of GLUT1 to the plasma membrane (PM) (21).
(C). Distinct from WTp53-mediated indirect regulation on metabolism which is mainly mediated by its downstream target genes (**Figure 1A** and **Table 1A**), MTp53 gains novel abilities to interact with a variety of proteins, particularly transcription factors, to drive tumor-associated metabolic alterations (**Figure 2C** and **Table 2C**). Examples of metabolic genes activated by MTp53 encompass *PLA2G16*, encoding a phospholipase catalyzing the formation of free fatty acids and lysophospholipids. The association of MTp53 with Kruppel-like factor 5 (KLF5) transactivates *PLA2G16*, which leads to increased glycolytic rate and accelerates tumor growth (32). Besides, enhanced expression of nucleotide metabolism genes (NMG) can be induced by cooperation between MTp53 and ETS proto-oncogene 2 transcription factor (ETS2), which is associated with poor prognosis in breast cancer patients (33). Gain-of-function of MTp53 also confers its ability to interact with the sterol regulatory element binding proteins (SREBPs) and be recruited to the sterol regulatory elements (SRE-1), which then induces genes encoding enzymes in the mevalonate pathway, contributing to the disrupted acinar formation in breast cancer cells (34). Later research further demonstrates that MTp53 exerts a differential regulation on NRF2 targets regardless of whether cells are under unstressed or oxidative stress conditions, where expression of genes upregulated by MTp53, including thioredoxin (TXN), thioredoxin reductase 1 (TXNRD1), proteasome 26S subunit ATPase 1 (PSMC1), and glutamate-cysteine ligase modifier subunit (GCLM), correlates with worse overall survival in breast cancer patients (35).

On the other hand, accumulated MTp53 proteins interfere with NRF2 activity, resulting in decreased expression of SLC7A11, a cystine/glutamate antiporter. Downregulation of SLC7A11 leads to GSH depletion and increased ROS, which sensitizes cancer cells to APR-246, a MTp53 reactivator (36). In another study, MTp53 is found to protect cancer cells confronted with oxidative stress from death through diminishing NRF2-mediated phase 2 ROS detoxifying enzymes, NAD(P)H quinone dehydrogenase (NQO1) and heme oxygenase 1 (HO-1) (37). Like the aforementioned, MTp53 differentially regulates NRF2 targets, in which metabolic genes are suppressed by MTp53 including HO-1, SLC7A11, and ATP binding cassette subfamily C member 3 (ABCC3) (35). Surprisingly, not only WTp53 binds to PGC-1α, MTp53 also interacts with PGC-1α; though this association exerts an inhibitory effect on PGC-1α. Basu et al. demonstrate that the codon 72 polymorphism in *TP53* impacts the binding and regulation of MTp53 to PGC-1α. The arginine 72 variant (R72) of MTp53, instead of the proline 72 variant (P72), shows decreased association with PGC-1α, resulting in increased PGC-1α function, enhanced OXPHOS, invasion, and metastasis (38).

## Targeting Mutant p53: Recent Advances and Future Perspectives

### Direct or Indirect?

Therapeutic strategies against tumors with p53 mutation are classified into direct and indirect approaches (42, 43). Direct targeting of MTp53 is attained by either restoring WTp53 function to MTp53 or depleting MTp53. Several MTp53 reactivators have been developed to refold MTp53 into the conformation of WTp53, prevent aggregation of MTp53 proteins, and restore DNA binding and transcriptional activity. In contrast, MTp53 destabilizers promote the degeneration of MTp53 and thus limit its expression, mainly through ubiquitin-proteasome degradation or autophagy-lysosome degradation pathways (44). Afterward, immunotherapies have emerged as promising strategies targeting tumors bearing MTp53 (45), including adoptive T cell therapy, the usage of antibody-drug conjugate (ADC) (46), and more recently, the development of the innovative bispecific antibody (BsAb) (47).

On the contrary, indirect approaches aim to disrupt connections between MTp53 and its synthetic lethal partners. Synthetic lethality is the concept that the simultaneous disturbance of two genes leads to cell death. In contrast, perturbation of an individual gene is tolerable, in which disruption of the two genes can be achieved by double mutations or single mutation of one gene combined with pharmacological inhibition of the other (48, 49). Due to the extensive influence of MTp53 on a variety of cellular processes and tumor development, synthetic lethality for cancer therapeutics against MTp53 is prospectively applicable to a wide range of cancers with *TP53* mutation, and thus, the identification of synthetic lethal genes to MTp53 becomes attractive. The development of synthetic lethal strategies targeting MTp53 provides more opportunities and therapeutic options for cancer patients with *TP53* mutation, which has advantages over approaches directly targeting MTp53, which are listed below.

(1). Synthetic lethality-based drugs are promising to broaden the strategies against cancers by their potential to overcome the major limitation of genetically targeted therapies. Not all cancer mutations are druggable, especially loss-of-function mutations (50, 51).
(2). It is quite challenging to develop MTp53 reactivators or inhibitors because of the diverse nature of MTp53 proteins. The structural diversity and the consequent distinct functional properties of every MTp53 make it practically infeasible to target all MTp53 with a single compound (42, 52). Synthetic lethal methods are more flexible because of the high dependency on MTp53-induced oncogenic effects rather than on MTp53 itself.
(3). MTp53 reactivators or inhibitors may induce severe toxicities and intolerable adverse effects. For example, MIRA-1, a MTp53 reactivator, could cause acute cytotoxicity to normal cells through induction of caspase-9-mediated apoptosis independent of p53 (53). APR-246 and COTI-2 are the only two drugs in clinical trials (42). Moreover, the relatively low-specificity inhibitors promoting degradation of MTp53, such as histone deacetylase inhibitors (HDACis), may elicit unfavorable adverse effects due to their widespread influence on normal cells (54).
(4). Restoration of WTp53 function to MTp53 might backfire because WTp53 has been paradoxically reported to favor tumor progression, particularly through metabolic regulation, which we highlight in this article. This notion is evident in a study that WTp53 potentiates glycolysis and decreases pyruvate uptake and thus OXPHOS in hepatocarcinoma (HCC) through inducing PUMA inhibition on mitochondrial pyruvate carrier (MPC) (55). Another study also substantiates that a low dosage of sulforaphane, the inducer of NRF2, could prevent apoptosis, promote proliferation, and enhance mitochondrial respiration in colorectal cancer (CRC) cells with WTp53 but not in p53-knockout CRC cells, suggesting a tumor-promoting role of WTp53 (56). Because of the potentially bi-faceted role of WTp53 in cancer, it is indispensable to ascertain the WTp53-mediated metabolic regulation and biological effects in different types of cancer.

### Synthetic Lethality With Metabolism

Several candidate synthetic lethal genes to p53 involved in various cellular processes, such as DNA repair, cell-cycle control, cell growth and proliferation, and metabolism, have been identified (57). Among these, we suggest that synthetic lethality targeting MTp53 with metabolism is the most potentially feasible for clinical application for the reasons stated below.

(1). In comparison to targeting MTp53 with metabolism, synthetic lethality with other cellular processes like DNA damage response and cell-cycle arrest may not only kill tumor cells but also damage normal cells, which could lead to severe adverse effects. For example, UCN01, an inhibitor of checkpoint kinase 1 (Chk1), the critical regulator of intra-S and G2/M checkpoints (58), has been halted for further clinical development due to the worse efficacy and the induction of unacceptable toxicities (59). Similarly, BI 2536, an inhibitor of polo-like kinase 1 (Plk1), which plays a crucial role in mitosis (60), exhibited a poor response rate and short half-life and could induce severe adverse events, leading to discontinued clinical progress (61).
(2). The highly intertwined relationships among different biomolecular processes contribute to the formation of metabolic networks, which interact with each other to form the complex construct of metabolism (62). Perturbations of the connected molecules could lead to disconnection of these pathways and breakdown of the metabolic networks, consequently destroying the resilience, which could cause cellular dysfunction and diseases (62). For tumor cells displaying high metabolic plasticity, the above features make it a potential treatment strategy to disrupt the crosstalk between the interconnected metabolic pathways by dual inhibition of these pathways with metabolic drugs or targeting compensatory mechanisms with a combination of drugs inhibiting global regulators and the respective compensatory bioenergetic pathways (63). Dual targeting of metabolic pathways as a therapeutic strategy has been demonstrated in the case of Lewis lung carcinoma (LLA), in which 2-deoxy-D-glucose (2-DG), a non-metabolizable analog of glucose that competitively binds to hexokinase and inhibits glycolysis (64), significantly enhances the antitumor activity of dichloroacetate (DCA), an inhibitor of pyruvate dehydrogenase kinase (PDK) that activates pyruvate dehydrogenase (PDH) and boosts OXPHOS activity (65, 66), accompanied with inhibition of glycolysis and increased cytotoxicity of tumor-infiltrating monocytes (67). In the case of p53 mutated breast cancer cells, 2-DG increases the sensitivity of tumor cells to metformin, which indirectly activates AMP-activated protein kinase (AMPK) by inhibiting complex I of the mitochondrial electron transport chain (68), indicating co-treatment of 2-DG and metformin may be an effective therapeutic strategy (69). Alternatively, a combination of phenformin, an AMPK activator, and 2-DG induces metabolic stress, suppresses tumor growth, and promotes degradation of MTp53 proteins (70), foreshadowing the promising future of inhibiting compensatory mechanisms accounting for the metabolic flexibility of tumor cells.
(3). Nowadays, there have been various metabolic drugs approved for clinical use in cancers and other diseases, such as 5-fluorouracil (5-FU) targeting nucleotide metabolism and metformin inhibiting mitochondrial function. Besides, several metabolic inhibitors are currently in cancer clinical trials (71), which are prospectively applicable in cancer treatments in the imminent future. Uncovering the MTp53-induced metabolic alterations in cancers would assist the utilization of these metabolic drugs in combination therapies or adjuvant therapies.

### Targeting p53-Mediated Indirect Regulation on Metabolism

In addition to targeting MTp53 with metabolism, mediators of MTp53-driven metabolic alterations are extremely potential synthetic lethal partners to MTp53. Specifically, the human genome comprises about 2% of protein-coding genes, leaving the vast majority to be non-coding RNAs (72). ncRNAs are critical regulators of many cellular processes, which indispensably coordinate the functional operation of complex networks in cells. Dysregulation of ncRNAs is detrimental, leading to pathological development like cancer (73). In viewing that p53 plays a pivotal role in regulating ncRNAs (12, 74), and that disturbance of ncRNAs contributes to cancer-associated metabolic reprogramming (75, 76), ncRNAs are expected to expand the synthetic lethal networks of MTp53 with metabolism. The examples below illustrate this point in detail.

As demonstrated in our previous work, MTp53 attenuates OXPHOS through disturbing the miR-200c-PCK2 axis, in which restoration of miR-200c rescues the decreased OXPHOS by MTp53 (14), implying therapeutic delivery of miR-200c through viral vectors or non-viral approaches, such as nanoparticles and liposomes (77), is a potential treatment strategy for intractable BLBC. It is noteworthy that restoring miR-200c expression not only boosts OXPHOS, but further nullifies MTp53-induced EMT and stemness, which may result from simultaneous activation of OXPHOS and direct inhibition of its downstream targets, ZEB1 and BMI1 (14). Likewise, miR-149-3p has been revealed to restore chemosensitivity of colorectal cancer cells through decreasing glycolytic activity *via* downregulating pyruvate dehydrogenase kinase 2 (PDK2), suggesting CRC patients with MTp53, in which the frequency of *TP53* mutation is around 40-50%, might benefit from combined employment of chemotherapy and miR-149-3p-based therapy (78). In addition to miRNAs, targeting lncRNAs synthetic lethal to MTp53 is also a potential therapeutic intervention. In the case aforementioned in the section of MTp53-mediated indirect regulation on metabolism, the lncRNA AGPG is upregulated by MTp53, which enhances glycolysis through preventing PFKFB3 from degradation (31). Interference of AGPG shows significantly inhibitory effects on tumor cells, providing implications for developing an RNA interference-based strategy.

For the same reason, the concept of expanding the synthetic lethality to MTp53 through targeting its indirect regulation on metabolism can apply to other mechanisms, like the signaling pathways modulated by MTp53 (**Figure 2B** and **Table 2B**), or the transcription factors associated with MTp53 (**Figure 2C** and **Table 2C**). Furthermore, in addition to metabolic drugs, numerous small-molecule targeted drugs and therapeutic monoclonal antibodies are FDA-approved for cancer treatment (79–81). Consequently, further investigation of molecular mechanisms accounting for MTp53-mediated metabolic reprogramming offers opportunities for patients with MTp53 tumors, in which these targeted drugs might be available in medical treatments.

Together, this evidence unequivocally indicates that targeting MTp53-mediated indirect regulation rather than directly targeting MTp53-driven metabolic alteration may elicit more powerful tumoricidal effects.

## Challenges on the Journey

Despite the accumulated myriad of research on p53 during the past four decades, a thorough understanding of the p53 regulatory network of cellular metabolism is still lacking. The progress of targeting MTp53 according to its regulation on cancer metabolism in practical clinical approaches remains stagnant, which could arise from the complexity of p53 and research limitations as following described.

(1). p53 regulation of metabolism is highly cell type-specific. A growing body of evidence has revealed that it is inappropriate to apply the general assumption of p53-regulated metabolism to all the types of cells, which is firmly supported by Kim et al. that WTp53 promotes glycolysis instead of OXPHOS in HCC (55). Furthermore, p53 regulates metabolism distinctly under stressed or unstressed conditions. For instance, p53 is demonstrated to increase OXPHOS in normoxia while decreasing OXPHOS in hypoxia in the cervix and breast cancer cells (82). As a result, the regulatory networks of p53 on cellular metabolism in different cells need to be more clearly defined.
(2). The exceedingly diverse nature of MTp53 proteins makes it a hurdle to precisely delineate MTp53-regulated metabolism in cancer cells. As shown in our previous study (14), stable expression of different MTp53 variants in normal mammary epithelial cells promotes glycolysis and suppresses mitochondrial respiration to varying extents. Erikson et al. also demonstrate that different hotspot mutations of p53 could have differential impacts on cellular metabolism like glycolysis and that endogenous and exogenous expression of even the same type of p53 mutation could exert opposite effects on OXPHOS (83).
(3). Murine cells and mouse models are frequently utilized in research exploring p53-regulated metabolism and the underlying molecular mechanisms; nevertheless, substantial differences in gene expression patterns and transcriptional regulatory programs have been observed between mice and humans (84). A more recent study corroborates that the p53 gene regulatory network (GRN) in mice differs from that in humans, in which the meta-analysis reveals extensive variation in p53-regulated gene expression profiles and high species-specificity of p53 transcriptional targets (85). This distinction can be exemplified by CPT1C, the brain isoform of the rate-limiting enzyme of FAO. CPT1C is generalized as a direct target of p53 (16, 86, 87); though, CPT1C is upregulated by p53 in mice but not in humans, reflecting the lack of p53 binding sites on human *CPT1C* promoter (85). In response to the differences in p53 GRN between mice and humans, more efforts are in need to confirm the metabolic roles of p53.
(4). Despite synthetic lethality targeting the mediators of MTp53-associated cancer metabolism portends a bright future for innumerable cancer patients with *TP53* mutation, the diverse nature of MTp53, the intracellular intricate regulatory networks accounting for the modulation of metabolism, and the extrinsic environmental factors determine together with the specific synthetic lethal partners to a specific type of MTp53, making identifying the synthetic lethal partners a quite challenging work. Once a synthetic lethal partner has been identified, the following concern is whether there are drugs specific for this synthetic lethal partner, or whether this synthetic lethal partner could be delivered to tumor sites or transported into tumor cells. The challenges of research on p53-mediated indirect regulation of metabolism are summarized below.
(a). The mechanism accounting for MTp53 indirect regulation of metabolism by dominant-negative effect over WTp53 (**Figure 2A** and **Table 2A**) may conform to multiple forms of MTp53, whereas the mechanisms underlying MTp53 indirect regulation of metabolism by gain-of-function (**Figures 2B, C** and **Tables 2B, C**) may not. Different types of MTp53 proteins might have distinct synthetic lethal partners; therefore, more efforts are required to identify the particular synthetic lethal relationship between the mediators involved in MTp53-regulated cancer metabolism and the specific types of MTp53.
(b). The synthetic lethal partner in a cell type could cause distinct, even opposite effects in another. PGC-1α illustrates this point clearly. In ERBB2^+^ breast cancers, high expression of PGC-1α predicts poorer prognosis and is correlated with high expression of glutamine cluster, reflecting the increased expression of PGC-1α in ERBB2^+^ breast cancer cells potentiates glutamine metabolism and facilitates proliferation under low glucose and hypoxia (88). On the contrary, elevated expression of PGC-1α leads to increased FAO and TCA cycle, decreased glycolysis, and suppression of tumor growth and metastasis in prostate cancers (89).
(c). There might be no targeted drug for a specific synthetic lethal partner, or it is tricky to develop it. For example, to our knowledge, there is no specific inhibitor for PGC-1α currently, which is activated by decreased binding to R72-MTp53, contributing to migration, invasion, and metastasis (38).
(d). Although successes in miRNA-based anti-cancer therapy have been reported (90), such as the significantly reduced tumor burden and improved survival rate in *Kras*;*Trp53* mutant NSCLC mice administered combinatorial treatment of miR-34a and let-7b using NOV340 liposomal nanoparticles (91), there are still challenges remained to be overcome in the clinical development of miRNA therapeutics. Maintaining stability and integrity of miRNA mimics or antagonists in the circulation, determining suitable dosages, delivering systems, and administration routes, increasing specificity and efficient penetration into tumors, as well as minimizing immunotoxicity and off-target effects all are issues needed to be concerned (90, 92).

In addition to the existing limitations to research on p53 regulatory networks of cellular metabolism, the links between p53-mediated cellular metabolism and p53-induced biological functions remain largely unknown since few studies have comprehensively deciphered the contribution of p53-regulated metabolic phenotype to tumor progression or tumor suppression. Deregulated metabolism has been designated as an emerging hallmark of cancer in the last decade (93), which implies uncovering the biological effects of metabolic alterations induced by p53 and the underlying molecular mechanisms is of great importance for designing the optimized treatment strategies. Like those mentioned above, our previous study reveals that MTp53 facilitates cancer stemness through attenuating OXPHOS by disturbance of the miR-200c-PCK2 axis (14), which fully addresses the molecular mechanism accounting for the connection between MTp53-induced metabolic alteration and traits of cancer. Notably, restoring miR-200c expression either in normal mammary epithelial cells overexpressing p53 mutants or in BLBC cells harboring endogenous MTp53 not only counteracts MTp53-induced EMT and stemness, but also recovers the decreased OXPHOS activity, foretelling the promising future of treating cancers with p53 mutation by modulating the mediators of metabolic reprogramming indirectly driven by MTp53. Moreover, metabostemness, describing cellular metabotypes as the driver to redirect normal cancer cells to less-differentiated cancer stem cell (CSC) states (94), interprets the significance of connecting metabolic features to traits of cancer cells, which opens a brand new way to cure cancer patients by circumventing resistance to therapies, metastasis, and tumor recurrence caused by CSCs (95) based on their metabolic dependencies.

## Summary

Overall, in addition to activating or repressing target genes transcriptionally, p53 regulates metabolism-related gene expression through indirect mechanisms as well, either by regulating direct transcriptional targets, associating with other transcription factors, or modulating signaling pathways. The notion that perturbations of p53 regulatory networks of cellular metabolism are in relation to tumor initiation and progression accentuates the crucial role of p53 in shaping cancer-associated metabolic phenotypes. Besides, *TP53*, as the most frequently mutated tumor suppressor gene in cancers, renders MTp53 promising for treatments of p53 mutated tumors. The diversity of MTp53 proteins and the unfavorable non-specific toxicities of MTp53 reactivators or inhibitors to normal tissues not only make it challenging to develop drugs directly targeting MTp53, but also impede the entrance of these drugs into clinical trials. Focusing on the synthetic lethal partners to MTp53 with metabolism, especially targeting the mediators involved in MTp53-driven metabolic reprogramming, might help broaden the synthetic lethal networks of MTp53. This would provide more opportunities and treatment options for cancer patients with MTp53, avoid or alleviate the off-target effects or severe adverse events, and forge the clinical application due to the existence of various metabolic and targeted drugs with FDA approval. Regardless of the substantial literature on p53 and its regulation of cellular metabolism, the metabolic roles of p53 and the mechanistic relationships between p53 and p53-mediated metabotypes are still ambiguous. Several limitations should be taken into consideration when we devote ourselves to investigating the metabolic networks of p53: (1) cell type-specificity of p53; (2) the highly diverse nature of MTp53; (3) differences in p53 GRN between mice and humans. Furthermore, challenges of research concentrating on p53-mediated indirect regulation on metabolism, include: (1) different MTp53 proteins might have distinct synthetic lethal partners; (2) synthetic lethal partner-exerted effects might be cell type-specific; (3) there might be no targeted drugs for a specific synthetic lethal partner; and (4) difficulties in the clinical development of ncRNA-based therapies, should be considered and kept in mind. Further research is also desired to unveil the biological effects of p53-associated metabolic activities, which is particularly essential for elucidating the contribution of p53-induced metabolic changes to the onset and malignancy of cancers, providing important implications for the development of prevention and treatment strategies.

## Author Contributions

C-YW and C-HC wrote the manuscript. All authors contributed to the article and approved the submitted version.

## Funding

This work was financially supported in part by the following: Ministry of Science and Technology (109-2628-B-009-004 to C-HC). The “Smart Platform of Dynamic Systems Biology for Therapeutic Development” and “Center for Intelligent Drug Systems and Smart Bio-devices (IDS^2^B) from The Featured Areas Research Center Program” within the framework of the Higher Education Sprout Project of the Ministry of Education (MOE) in Taiwan.

## Conflict of Interest

The authors declare that the research was conducted in the absence of any commercial or financial relationships that could be construed as a potential conflict of interest.

## Publisher’s Note

All claims expressed in this article are solely those of the authors and do not necessarily represent those of their affiliated organizations, or those of the publisher, the editors and the reviewers. Any product that may be evaluated in this article, or claim that may be made by its manufacturer, is not guaranteed or endorsed by the publisher.

## References

[1] Boutelle AM Attardi LD. P53 and Tumor Suppression: It Takes a Network. Trends Cell Biol (2021) 31(4):298–310. doi: 10.1016/j.tcb.2020.12.011 33518400 PMC7954925

[2] Levine AJ. P53: 800 Million Years of Evolution and 40 Years of Discovery. Nat Rev Cancer (2020) 20(8):471–80. doi: 10.1038/s41568-020-0262-1 32404993

[3] Joerger AC Fersht AR. The P53 Pathway: Origins, Inactivation in Cancer, and Emerging Therapeutic Approaches. Annu Rev Biochem (2016) 85:375–404. doi: 10.1146/annurev-biochem-060815-014710 27145840

[4] Leroy B Anderson M Soussi T. TP53 Mutations in Human Cancer: Database Reassessment and Prospects for the Next Decade. Hum Mutat (2014) 35(6):672–88. doi: 10.1002/humu.22552 24665023

[5] Brosh R Rotter V. When Mutants Gain New Powers: News From the Mutant P53 Field. Nat Rev Cancer (2009) 9(10):701–13. doi: 10.1038/nrc2693 19693097

[6] Baugh EH Ke H Levine AJ Bonneau RA Chan CS. Why Are There Hotspot Mutations in the TP53 Gene in Human Cancers? Cell Death Differ (2018) 25(1):154–60. doi: 10.1038/cdd.2017.180 PMC5729536 29099487

[7] Weisz L Oren M Rotter V. Transcription Regulation by Mutant P53. Oncogene (2007) 26(15):2202–11. doi: 10.1038/sj.onc.1210294 17401429

[8] Soussi T. P53 Alterations in Human Cancer: More Questions Than Answers. Oncogene (2007) 26(15):2145–56. doi: 10.1038/sj.onc.1210280 17401423

[9] Kim E Deppert W. Interactions of Mutant P53 With DNA: Guilt by Association. Oncogene (2007) 26(15):2185–90. doi: 10.1038/sj.onc.1210312 17401427

[10] Alvarado-Ortiz E de la Cruz-López K Becerril-Rico J Sarabia-Sánchez M Ortiz-Sánchez E García-Carrancá A. Mutant P53 Gain-Of-Function: Role in Cancer Development, Progression, and Therapeutic Approaches. Front Cell Dev Biol (2020) 8:607670–0. doi: 10.3389/fcell.2020.607670 PMC7905058 33644030

[11] Pfister NT Fomin V Regunath K Zhou JY Zhou W Silwal-Pandit L. Mutant P53 Cooperates With the SWI/SNF Chromatin Remodeling Complex to Regulate VEGFR2 in Breast Cancer Cells. Genes Dev (2015) 29(12):1298–315. doi: 10.1101/gad.263202.115 PMC4495400 26080815

[12] Di Agostino S. The Impact of Mutant P53 in the Non-Coding RNA World. Biomolecules (2020) 10(3):472. doi: 10.3390/biom10030472 PMC7175150 32204575

[13] Vousden KH Ryan KM. P53 and Metabolism. Nat Rev Cancer (2009) 9(10):691–700. doi: 10.1038/nrc2715 19759539

[14] Chao C-H Wang C-Y Wang C-H Chen T-W Hsu H-Y Huang H-W. Mutant P53 Attenuates Oxidative Phosphorylation and Facilitates Cancer Stemness Through Downregulating miR-200c–PCK2 Axis in Basal-Like Breast Cancer. Mol Cancer Res (2021) 19(11):1900–16. doi: 10.1158/1541-7786.MCR-21-0098 34312289

[15] Yu S Meng S Xiang M Ma H. Phosphoenolpyruvate Carboxykinase in Cell Metabolism: Roles and Mechanisms Beyond Gluconeogenesis. Mol Metab (2021) 53:101257–7. doi: 10.1016/j.molmet.2021.101257 PMC8190478 34020084

[16] Lacroix M Riscal R Arena G Linares LK Le Cam L. Metabolic Functions of the Tumor Suppressor P53: Implications in Normal Physiology, Metabolic Disorders, and Cancer. Mol Metab (2020) 33:2–22. doi: 10.1016/j.molmet.2019.10.002 31685430 PMC7056927

[17] Mathupala SP Heese C Pedersen PL. Glucose Catabolism in Cancer Cells: The Type II Hexokinase Promoter Contains Functionally Active Response Elements for the Tumor Suppressor P53. J Biol Chem (1997) 272(36):22776–80. doi: 10.1074/jbc.272.36.22776 9278438

[18] Kim H-R Roe J-S Lee J-E Cho E-J Youn H-D. P53 Regulates Glucose Metabolism by miR-34a. Biochem Biophysl Res Commun (2013) 437(2):225–31. doi: 10.1016/j.bbrc.2013.06.043 23796712

[19] Wang L Xiong H Wu F Zhang Y Wang J Zhao L. Hexokinase 2-Mediated Warburg Effect Is Required for PTEN-And P53-Deficiency-Driven Prostate Cancer Growth. Cell Rep (2014) 8(5):1461–74. doi: 10.1016/j.celrep.2014.07.053 PMC4360961 25176644

[20] Schwartzenberg-Bar-Yoseph F Armoni M Karnieli E. The Tumor Suppressor P53 Down-Regulates Glucose Transporters GLUT1 and GLUT4 Gene Expression. Cancer Res (2004) 64(7):2627–33. doi: 10.1158/0008-5472.CAN-03-0846 15059920

[21] Zhang C Liu J Liang Y Wu R Zhao Y Hong X. Tumour-Associated Mutant P53 Drives the Warburg Effect. Nat Commun (2013) 4(1):1–15. doi: 10.1038/ncomms3935 PMC3969270 24343302

[22] Kim H-R Roe J-S Lee J-E Hwang I-Y Cho E-J Youn H-D. A P53-Inducible microRNA-34a Downregulates Ras Signaling by Targeting IMPDH. Biochem Biophys Res Commun (2012) 418(4):682–8. doi: 10.1016/j.bbrc.2012.01.077 22301190

[23] Azar S Udi S Drori A Hadar R Nemirovski A Vemuri KV. Reversal of Diet-Induced Hepatic Steatosis by Peripheral CB1 Receptor Blockade in Mice Is P53/miRNA-22/Sirt1/Pparα Dependent. Mol Metab (2020) 42:101087–7. doi: 10.1016/j.molmet.2020.101087 PMC7563015 32987186

[24] Liao M Liao W Xu N Li B Liu F Zhang S. LncRNA EPB41L4A-AS1 Regulates Glycolysis and Glutaminolysis by Mediating Nucleolar Translocation of HDAC2. EBioMedicine (2019) 41:200–13. doi: 10.1016/j.ebiom.2019.01.035 PMC6444057 30796006

[25] Aquilano K Baldelli S Pagliei B Cannata SM Rotilio G Ciriolo MR. P53 Orchestrates the PGC-1α-Mediated Antioxidant Response Upon Mild Redox and Metabolic Imbalance. Antioxid Redox Signal (2013) 18(4):386–99. doi: 10.1089/ars.2012.4615 PMC3526895 22861165

[26] Zhang P Tu B Wang H Cao Z Tang M Zhang C. Tumor Suppressor P53 Cooperates With SIRT6 to Regulate Gluconeogenesis by Promoting FoxO1 Nuclear Exclusion. Proc Natl Acad Sci (2014) 111(29):10684–9. doi: 10.1073/pnas.1411026111 PMC4115576 25009184

[27] Dhar SK Xu Y Chen Y Clair DKS. Specificity Protein 1-Dependent P53-Mediated Suppression of Human Manganese Superoxide Dismutase Gene Expression. J Biol Chem (2006) 281(31):21698–709. doi: 10.1074/jbc.M601083200 PMC2640468 16740634

[28] Sen N Satija YK Das S. PGC-1α, a Key Modulator of P53, Promotes Cell Survival Upon Metabolic Stress. Mol Cell (2011) 44(4):621–34. doi: 10.1016/j.molcel.2011.08.044 22099309

[29] Kawauchi K Araki K Tobiume K Tanaka N. P53 Regulates Glucose Metabolism Through an IKK-NF-κB Pathway and Inhibits Cell Transformation. Nat Cell Biol (2008) 10(5):611–8. doi: 10.1038/ncb1724 18391940

[30] Deng X Li Y Gu S Chen Y Yu B Su J. P53 Affects PGC-1α Stability Through AKT/GSK-3β to Enhance Cisplatin Sensitivity in Non-Small Cell Lung Cancer. Front Oncol (2020) 10:1252–2. doi: 10.3389/fonc.2020.01252 PMC7471661 32974127

[31] Liu J Liu Z-X Wu Q-N Lu Y-X Wong C-W Miao L. Long Noncoding RNA AGPG Regulates PFKFB3-Mediated Tumor Glycolytic Reprogramming. Nat Commun (2020) 11(1):1–16. doi: 10.1038/s41467-020-15112-3 32198345 PMC7083971

[32] Xia W Bai H Deng Y Yang Y. PLA2G16 Is a Mutant P53/KLF5 Transcriptional Target and Promotes Glycolysis of Pancreatic Cancer. J Cell Mol Med (2020) 24(21):12642–55. doi: 10.1111/jcmm.15832 PMC7686977 32985124

[33] Kollareddy M Dimitrova E Vallabhaneni KC Chan A Le T Chauhan KM. Regulation of Nucleotide Metabolism by Mutant P53 Contributes to Its Gain-Of-Function Activities. Nat Commun (2015) 6(1):1–13. doi: 10.1038/ncomms8389 PMC4467467 26067754

[34] Moon RR-B Barsotti A Chicas A Li W Polotskaia10 A Bissell11 MJ. Mutant P53 Disrupts Mammary Acinar Morphogenesis *Via* the Mevalonate Pathway. Cell (2012) 148(1-2):244–58. doi: 10.1016/j.cell.2011.12.017 PMC3511889 22265415

[35] Lisek K Campaner E Ciani Y Walerych D Del Sal G. Mutant P53 Tunes the NRF2-Dependent Antioxidant Response to Support Survival of Cancer Cells. Oncotarget (2018) 9(29):20508–23. doi: 10.18632/oncotarget.24974 PMC5945496 29755668

[36] Liu DS Duong CP Haupt S Montgomery KG House CM Azar WJ. Inhibiting the System Xc^–^/Glutathione Axis Selectively Targets Cancers With Mutant-P53 Accumulation. Nat Commun (2017) 8(1):1–14. doi: 10.1038/ncomms14844 28348409 PMC5379068

[37] Kalo E Kogan-Sakin I Solomon H Bar-Nathan E Shay M Shetzer Y. Mutant P53^R273H^ Attenuates the Expression of Phase 2 Detoxifying Enzymes and Promotes the Survival of Cells With High Levels of Reactive Oxygen Species. J Cell Sci (2012) 125(22):5578–86. doi: 10.1242/jcs.106815 22899716

[38] Basu S Gnanapradeepan K Barnoud T Kung C-P Tavecchio M Scott J. Mutant P53 Controls Tumor Metabolism and Metastasis by Regulating PGC-1α. Genes Dev (2018) 32(3-4):230–43. doi: 10.1101/gad.309062.117 PMC5859965 29463573

[39] Chang C-J Chao C-H Xia W Yang J-Y Xiong Y Li C-W. P53 Regulates Epithelial–Mesenchymal Transition and Stem Cell Properties Through Modulating miRNAs. Nat Cell Biol (2011) 13(3):317–23. doi: 10.1038/ncb2173 PMC3075845 21336307

[40] Jeninga EH Schoonjans K Auwerx J. Reversible Acetylation of PGC-1: Connecting Energy Sensors and Effectors to Guarantee Metabolic Flexibility. Oncogene (2010) 29(33):4617–24. doi: 10.1038/onc.2010.206 PMC3843141 20531298

[41] Dhar SK Clair DKS. Manganese Superoxide Dismutase Regulation and Cancer. Free Radic Biol Med (2012) 52(11-12):2209–22. doi: 10.1016/j.freeradbiomed.2012.03.009 22561706

[42] Hu J Cao J Topatana W Juengpanich S Li S Zhang B. Targeting Mutant P53 for Cancer Therapy: Direct and Indirect Strategies. J Hematol Oncol (2021) 14(1):1–19. doi: 10.1186/s13045-021-01169-0 34583722 PMC8480024

[43] Zhang C Liu J Xu D Zhang T Hu W Feng Z. Gain-Of-Function Mutant P53 in Cancer Progression and Therapy. J Mol Cell Biol (2020) 12(9):674–87. doi: 10.1093/jmcb/mjaa040 PMC7749743 32722796

[44] Xu Z Wu W Yan H Hu Y He Q Luo P. Regulation of P53 Stability as a Therapeutic Strategy for Cancer. Biochem Pharmacol (2021) 185:114407–04407. doi: 10.1016/j.bcp.2021.114407 33421376

[45] Chasov V Zaripov M Mirgayazova R Khadiullina R Zmievskaya E Ganeeva I. Promising New Tools for Targeting P53 Mutant Cancers: Humoral and Cell-Based Immunotherapies. Front Immunol (2021) 12:707734–4. doi: 10.3389/fimmu.2021.707734 PMC8411701 34484205

[46] Low L Goh A Koh J Lim S Wang C-I. Targeting Mutant P53-Expressing Tumours With a T Cell Receptor-Like Antibody Specific for a Wild-Type Antigen. Nat Commun (2019) 10(1):1–14. doi: 10.1038/s41467-019-13305-z 31772160 PMC6879612

[47] Yang C Lou G Jin W. The Arsenal of TP53 Mutants Therapies: Neoantigens and Bispecific Antibodies. Signal Transduct Target Ther (2021) 6(1):1–2. doi: 10.1038/s41392-021-00635-y 34083505 PMC8175382

[48] O'Neil NJ Bailey ML Hieter P. Synthetic Lethality and Cancer. Nat Rev Genet (2017) 18(10):613–23. doi: 10.1038/nrg.2017.47 28649135

[49] Topatana W Juengpanich S Li S Cao J Hu J Lee J. Advances in Synthetic Lethality for Cancer Therapy: Cellular Mechanism and Clinical Translation. J Hematol Oncol (2020) 13(1):1–22. doi: 10.1186/s13045-020-00956-5 32883316 PMC7470446

[50] Huang A Garraway LA Ashworth A Weber B. Synthetic Lethality as an Engine for Cancer Drug Target Discovery. Nat Rev Drug Discovery (2020) 19(1):23–38. doi: 10.1038/s41573-019-0046-z 31712683

[51] Li S Topatana W Juengpanich S Cao J Hu J Zhang B. Development of Synthetic Lethality in Cancer: Molecular and Cellular Classification. Signal Transduct Target Ther (2020) 5(1):1–14. doi: 10.1038/s41392-020-00358-6 33077733 PMC7573576

[52] Sabapathy K Lane DP. Therapeutic Targeting of P53: All Mutants Are Equal, But Some Mutants Are More Equal Than Others. Nat Rev Clin Oncol (2018) 15(1):13–30. doi: 10.1038/nrclinonc.2017.151 28948977

[53] Bou-Hanna C Jarry A Lode L Schmitz I Schulze-Osthoff K Kury S. Acute Cytotoxicity of MIRA-1/NSC19630, a Mutant P53-Reactivating Small Molecule, Against Human Normal and Cancer Cells *Via* a Caspase-9-Dependent Apoptosis. Cancer Lett (2015) 359(2):211–7. doi: 10.1016/j.canlet.2015.01.014 25617798

[54] Borrero LJH El-Deiry WS. Tumor Suppressor P53: Biology, Signaling Pathways, and Therapeutic Targeting. Biochim Biophys Acta Rev Cancer (2021) 1876(1):188556. doi: 10.1016/j.bbcan.2021.188556 33932560 PMC8730328

[55] Kim J Yu L Chen W Xu Y Wu M Todorova D. Wild-Type P53 Promotes Cancer Metabolic Switch by Inducing PUMA-Dependent Suppression of Oxidative Phosphorylation. Cancer Cell (2019) 35(2):191–203. doi: 10.1016/j.ccell.2018.12.012 30712844

[56] Gwon Y Oh J Kim J-S. Sulforaphane Induces Colorectal Cancer Cell Proliferation Through Nrf2 Activation in a P53-Dependent Manner. Appl Biol Chem (2020) 63(1):1–11. doi: 10.1186/s13765-020-00578-y

[57] Wang X Simon R. Identification of Potential Synthetic Lethal Genes to P53 Using a Computational Biology Approach. BMC Med Genomics (2013) 6(1):1–10. doi: 10.1186/1755-8794-6-30 24025726 PMC3847148

[58] Neizer-Ashun F Bhattacharya R. Reality CHEK: Understanding the Biology and Clinical Potential of CHK1. Cancer Lett (2021) 497:202–11. doi: 10.1016/j.canlet.2020.09.016 32991949

[59] Morandell S Yaffe MB. Exploiting Synthetic Lethal Interactions Between DNA Damage Signaling, Checkpoint Control, and P53 for Targeted Cancer Therapy. Prog Mol Biol Transl Sci (2012) 110:289–314. doi: 10.1016/B978-0-12-387665-2.00011-0 22749150

[60] Lee S-Y Jang C Lee K-A. Polo-Like Kinases (Plks), a Key Regulator of Cell Cycle and New Potential Target for Cancer Therapy. Dev Reprod (2014) 18(1):65–71. doi: 10.12717/DR.2014.18.1.065 25949173 PMC4282265

[61] Vose JM Friedberg JW Waller EK Cheson BD Juvvigunta V Fritsch H. The Plk1 Inhibitor BI 2536 in Patients With Refractory or Relapsed Non-Hodgkin Lymphoma: A Phase I, Open-Label, Single Dose-Escalation Study. Leuk Lymphoma (2013) 54(4):708–13. doi: 10.3109/10428194.2012.729833 22978685

[62] Gómez-Romero L López-Reyes K Hernández-Lemus E. The Large Scale Structure of Human Metabolism Reveals Resilience *Via* Extensive Signaling Crosstalk. Front Physiol (2020) 11:588012–2. doi: 10.3389/fphys.2020.588012 PMC7772240 33391012

[63] McGuirk S Audet-Delage Y St-Pierre J. Metabolic Fitness and Plasticity in Cancer Progression. Trends Cancer (2020) 6(1):49–61. doi: 10.1016/j.trecan.2019.11.009 31952781

[64] Bere J Jonnalagadda ND Kappari L Karangula J Boggula N Kappari V. 2-Deoxy-D-Glucose: An Update Review. J Innov Dev Pharm Tech Sci(JIDPTS) (2021) 4(5):68–78.

[65] Michelakis E Webster L Mackey J. Dichloroacetate (DCA) as a Potential Metabolic-Targeting Therapy for Cancer. Br J Cancer (2008) 99(7):989–94. doi: 10.1038/sj.bjc.6604554 PMC2567082 18766181

[66] Tataranni T Piccoli C. Dichloroacetate (DCA) and Cancer: An Overview Towards Clinical Applications. Oxid Med Cell Longev (2019) 2019:8201079–8201079. doi: 10.1155/2019/8201079 31827705 PMC6885244

[67] Pyaskovskaya O Kolesnik D Fedorchuk A Prochorova I Solyanik G. 2-Deoxy-D-Glucose Enhances Dichloroacetate Antitumor Action Against Lewis Lung Carcinoma. Exp Oncol (2016) 38(3):176–80. doi: 10.31768/2312-8852.2016.38(3):176-180 27685525

[68] Pernicova I Korbonits M. Metformin—Mode of Action and Clinical Implications for Diabetes and Cancer. Nat Rev Endocrinol (2014) 10(3):143–56. doi: 10.1038/nrendo.2013.256 24393785

[69] Rajh M Dolinar K Miš K Pavlin M Pirkmajer S. Medium Renewal Blocks Anti-Proliferative Effects of Metformin in Cultured MDA-MB-231 Breast Cancer Cells. PloS One (2016) 11(5):e0154747–e0154747. doi: 10.1371/journal.pone.0154747 27135408 PMC4852933

[70] Jung CL Mun H Jo S-Y Oh J-H Lee C Choi E-K. Suppression of Gain-Of-Function Mutant P53 With Metabolic Inhibitors Reduces Tumor Growth *In Vivo* . Oncotarget (2016) 7(47):77664–682. doi: 10.18632/oncotarget.12758 PMC5363612 27765910

[71] Stine ZE Schug ZT Salvino JM Dang CV. Targeting Cancer Metabolism in the Era of Precision Oncology. Nat Rev Drug Discovery (2022) 21(2):141–62. doi: 10.1038/s41573-021-00339-6 PMC8641543 34862480

[72] Wright MW Bruford EA. Naming'junk': Human Non-Protein Coding RNA (ncRNA) Gene Nomenclature. Hum Genomics (2011) 5(2):1–9. doi: 10.1186/1479-7364-5-2-90 PMC3051107 21296742

[73] Anastasiadou E Jacob LS Slack FJ. Non-Coding RNA Networks in Cancer. Nat Rev Cancer (2018) 18(1):5–18. doi: 10.1038/nrc.2017.99 29170536 PMC6337726

[74] Chen S Thorne RF Zhang XD Wu M Liu L. Non-Coding RNAs, Guardians of the P53 Galaxy. Semin Cancer Biol (2021) 75:72–83. doi: 10.1016/j.semcancer.2020.09.002 32927018

[75] Beltrán-Anaya FO Cedro-Tanda A Hidalgo-Miranda A Romero-Cordoba SL. Insights Into the Regulatory Role of Non-Coding RNAs in Cancer Metabolism. Front Physiol (2016) 7:342–2. doi: 10.3389/fphys.2016.00342 PMC4976125 27551267

[76] Lin X Wu Z Hu H Luo M-L Song E. Non-Coding RNAs Rewire Cancer Metabolism Networks. Semin Cancer Biol (2021) 75:116–26. doi: 10.1016/j.semcancer.2020.12.019 33421618

[77] Forterre A Komuro H Aminova S Harada M. A Comprehensive Review of Cancer MicroRNA Therapeutic Delivery Strategies. Cancers (2020) 12(7):1852. doi: 10.3390/cancers12071852 PMC7408939 32660045

[78] Liang Y Hou L Li L Li L Zhu L Wang Y. Dichloroacetate Restores Colorectal Cancer Chemosensitivity Through the P53/Mir-149-3p/PDK2-Mediated Glucose Metabolic Pathway. Oncogene (2020) 39(2):469–85. doi: 10.1038/s41388-019-1035-8 PMC6949190 31597953

[79] Zhong L Li Y Xiong L Wang W Wu M Yuan T. Small Molecules in Targeted Cancer Therapy: Advances, Challenges, and Future Perspectives. Signal Transduct Target Ther (2021) 6(1):1–48. doi: 10.1038/s41392-021-00572-w 34054126 PMC8165101

[80] Lu R-M Hwang Y-C Liu I-J Lee C-C Tsai H-Z Li H-J. Development of Therapeutic Antibodies for the Treatment of Diseases. J BioMed Sci (2020) 27(1):1–30. doi: 10.1186/s12929-019-0592-z 31894001 PMC6939334

[81] Ke X Shen L. Molecular Targeted Therapy of Cancer: The Progress and Future Prospect. Front Lab Med (2017) 1(2):69–75. doi: 10.1016/j.flm.2017.06.001

[82] Hernández-Reséndiz I Román-Rosales A García-Villa E López-Macay A Pineda E Saavedra E. Dual Regulation of Energy Metabolism by P53 in Human Cervix and Breast Cancer Cells. Biochim Biophys Acta Mol Cell Res (2015) 1853(12):3266–78. doi: 10.1016/j.bbamcr.2015.09.033 26434996

[83] Eriksson M Ambroise G Ouchida AT Lima Queiroz A Smith D Gimenez-Cassina A. Effect of Mutant P53 Proteins on Glycolysis and Mitochondrial Metabolism. Mol Cell Biol (2017) 37(24):e00328–17. doi: 10.1128/MCB.00328-17 PMC5705820 28993478

[84] Yue F Cheng Y Breschi A Vierstra J Wu W Ryba T. A Comparative Encyclopedia of DNA Elements in the Mouse Genome. Nature (2014) 515(7527):355–64. doi: 10.1038/nature13992 PMC4266106 25409824

[85] Fischer M. Conservation and Divergence of the P53 Gene Regulatory Network Between Mice and Humans. Oncogene (2019) 38(21):4095–109. doi: 10.1038/s41388-019-0706-9 PMC6755996 30710145

[86] Reilly PT Mak TW. Molecular Pathways: Tumor Cells Co-Opt the Brain-Specific Metabolism Gene CPT1C to Promote Survival. Clin Cancer Res (2012) 18(21):5850–5. doi: 10.1158/1078-0432.CCR-11-3281 22952346

[87] Goldstein I Rotter V. Regulation of Lipid Metabolism by P53–Fighting Two Villains With One Sword. Trends Endocrinol Metab (2012) 23(11):567–75. doi: 10.1016/j.tem.2012.06.007 22819212

[88] McGuirk S Gravel S-P Deblois G Papadopoli DJ Faubert B Wegner A. PGC-1α Supports Glutamine Metabolism in Breast Cancer. Cancer Metab (2013) 1(1):1–11. doi: 10.1186/2049-3002-1-22 24304688 PMC4178216

[89] Torrano V Valcarcel-Jimenez L Cortazar AR Liu X Urosevic J Castillo-Martin M. The Metabolic Co-Regulator PGC-1α Suppresses Prostate Cancer Metastasis. Nat Cell Biol (2016) 18(6):645–56. doi: 10.1038/ncb3357 PMC4884178 27214280

[90] Reda El Sayed S Cristante J Guyon L Denis J Chabre O Cherradi N. MicroRNA Therapeutics in Cancer: Current Advances and Challenges. Cancers (2021) 13(11):2680. doi: 10.3390/cancers13112680 34072348 PMC8198729

[91] Kasinski AL Kelnar K Stahlhut C Orellana E Zhao J Shimer E. A Combinatorial MicroRNA Therapeutics Approach to Suppressing Non-Small Cell Lung Cancer. Oncogene (2015) 34(27):3547–55. doi: 10.1038/onc.2014.282 PMC4345154 25174400

[92] Chen Y Gao D-Y Huang L. *In Vivo* Delivery of miRNAs for Cancer Therapy: Challenges and Strategies. Adv Drug Del Rev (2015) 81:128–41. doi: 10.1016/j.addr.2014.05.009 PMC5009470 24859533

[93] Hanahan D Weinberg RA. Hallmarks of Cancer: The Next Generation. Cell (2011) 144(5):646–74. doi: 10.1016/j.cell.2011.02.013 21376230

[94] Menendez JA Alarcón T. Metabostemness: A New Cancer Hallmark. Front Oncol (2014) 4:262–2. doi: 10.3389/fonc.2014.00262 PMC4179679 25325014

[95] Yang L Shi P Zhao G Xu J Peng W Zhang J. Targeting Cancer Stem Cell Pathways for Cancer Therapy. Signal Transduct Target Ther (2020) 5(1):1–35. doi: 10.1038/s41392-020-0110-5 32296030 PMC7005297

